# Oncocytoma of Palate: A Case Report, with an Emphasis on Immunohistochemistry 

**DOI:** 10.30476/dentjods.2025.105376.2591

**Published:** 2026-03-01

**Authors:** Shubhangi Mani, Amit Mani, Vandana Pandey Tripathi, Manas Bajpai, Shyam Reddy Karakalla

**Affiliations:** 1 Dept. of Orthodontics and Dentofacial Orthopaedics, Rural Dental College, Loni, India.; 2 Dept. of Periodontology, Rural Dental College Loni, India.; 3 Dept. of Pedodontics and Preventive Dentistry Government Dental College, Mumbai, India.; 4 Dept. of Oral Pathology and Microbiology, Rural Dental College, Loni, India.; 5 Kamineni Hospital, Vijayawada (Andhra Pradesh), India.

**Keywords:** Oncocytoma, Minor salivary gland, Salivary gland tumor, Immunohistochemistry

## Abstract

Oncocytomas are benign tumors of salivary glands, characterized histopathologically by large epithelial cells with bright and eosinophilic cytoplasm. They occur mostly in parotid glands, with only a small percentage of these tumors occurring in minor salivary glands of buccal, mucosa, palate, maxillary sinus, and tonsillar fossa. They occur primarily in people older than 50 years and generally have a female predilection. Few authors have suggested that oncocytomas of minor salivary glands are more aggressive in nature in comparison to their major salivary gland counterparts. An exhaustive literature review of English language literature revealed 20 cases of minor salivary glands; hence, the present case is 21st case of minor salivary gland oncocytoma. We report a case of palatal oncocytoma in a 31-year-old male which was provisionally diagnosed as pleomorphic adenoma and also describe the immunohistochemical characteristics of this rare tumor of minor salivary glands.

## Introduction

Oncocytoma was first reported in the salivary gland by Hamperl in 1931 [ 1 ]. The term ‘oncocytoma’ was coined by Jaffe in 1932 to designate a benign salivary gland tumor that chiefly occurs in the parotid gland. These tumors are histologically characterized by oncocytic cells lining the salivary ducts [ 2 ]. In 1932, Meza-Chavez proposed the term ‘oxyphilic granular cell adenoma’ owing to a diffuse granular appearance of the cytoplasm of tumor cells [ 3 ]. Intraoral oncocytomas clinically exhibit asymptomatic, solitary, and soft tissue growth of minor salivary glands, with less than 1% of tumors transforming into malignancy [ 4 ]. The diagnosis of oncocytomas is generally based on the histopathological picture; immunohistochemistry may be required for diagnostic confirmation. The purpose of this manuscript is to report an additional case of minor salivary gland oncocytoma, to review all the previously published cases and to discuss the immunohistochemical features of this rare tumor briefly.

## Case Presentation

**Figure 1:**
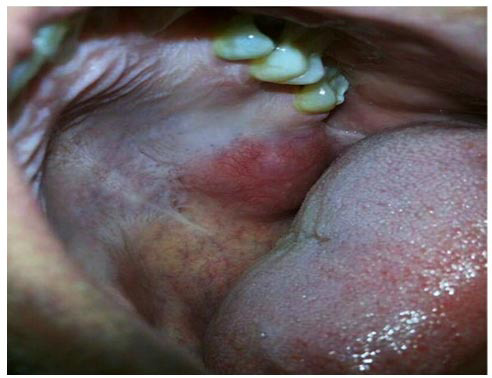
Clinical picture of the palatal growth

An otherwise healthy 31 years old male presented with a large painless mass on his upper left region of palate with 1 year duration. The mass was gradually increasing and reached its present size. The patient was having dysphagia and breathing difficulty due to the growth. Past medical history and family history were not consistent with the presenting symptom. Intraoral examination revealed a solitary ovoid growth involving the hard palate, extending anteroposteriorly from the palatal aspect of tooth #24 to the left maxillary tuberosity, measuring about 3×2cm. The color of the swelling was pink to slightly reddish (Figure 1). On palpation, the mass was soft to firm in consistency. No signs of discharge and secondary infection were found. 

**Figure 2:**
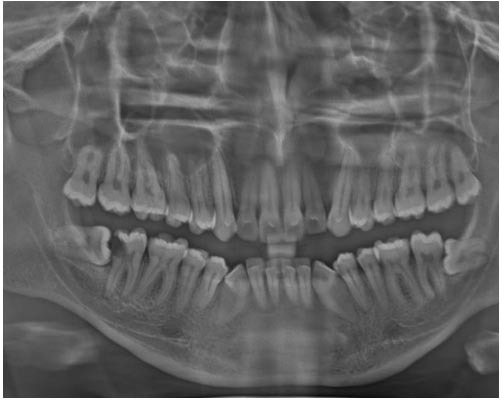
There was no evidence of bone involvement in panoramic radiograph

The panoramic radiograph confirmed that the lesion was peripheral and was not associated with the underlying bone (Figure 2). 

A provisional diagnosis of pleomorphic adenoma was given and differential diagnoses of fibroma, and ossifying fibroma were considered. The tumor was completely excised under local anesthesia, and the tissue was sent to the Department of Oral Pathology and Microbiology for microscopic evaluation. 

**Figure 3:**
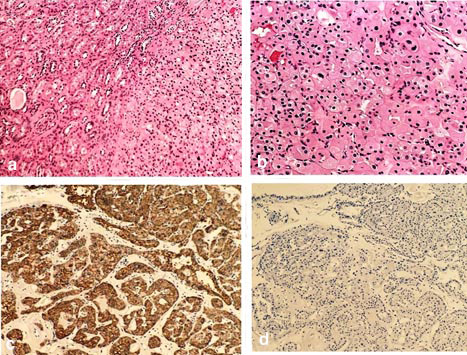
**a:** Sheets of polygonal cells with granular cytoplasm, separated by fibro - vascular stroma. (Hematoxylin and Eosin stain 10×), **b:** Polygonal neoplastic cells having centrally placed nucleus and abundant granular cytoplasm. (Hematoxylin and Eosin stain 40×), **c:** Tumor cells show positive expression for CK 7, **d:** The tumor cells show negative expression for CD -117 (10×)

Histopathological examination of the soft tissue section revealed a well-circumscribed tumor mass comprised of solid sheets of granular cells separated by the fibrous band (Figure 3a). Higher magnification exhibited the cells were polyhedral to round in shape with abundant granular and intense cytoplasm resembling oncocytes (Figure 3b). 

Immunohistochemical staining was carried out using CD 117 (c-KIT) and CK 7 markers. The tumor cells show positive expression for CK-7 (Figure 3c) and negative expression of CD -117, (Figure 3d) confirming the diagnosis of oncocytoma and ruling out the epithelium origin of the neoplasm. 

Based on histopathological and immunohistochemical findings, a final diagnosis of oncocytoma was given. The 1-year follow-up of the patient was uneventful.

## Discussion

Scaffer described oncocytic lesions as granular swollen cells in the ductal and acinar elements of the salivary gland [ 1 , 3 ]. The World Health Organization (WHO) in 1991 classified three different oncocytic entities including oncocytosis, oncocytoma and oncocytic carcinoma [ 3 - 6 ]. It is common agreement that a tumor to be called an oncocytoma should be exclusively comprised of oncocytes (Table 1). Oncocytomas are rare benign, solitary, asymptomatic salivary gland neoplasms representing less than 1% of all salivary gland tumors, usually seen in the 7th and 8th decades of life with slight female predilection [ 4 , 6 - 9 ]. Oncocytomas in intraoral minor salivary glands are exceedingly rare; only 20 cases have been reported to date [ 10 ]. Oncocytomas are believed to arise from epithelial cells undergoing metabolic changes, leading to an accumulation of mitochondria within the cytoplasm. This accumulation is responsible for the characteristic eosinophilic granular appearance under microscopic examination. While the exact etiology remains unknown, factors like aging, chronic inflammation, and potential genetic mutations are implicated [ 5 - 8 ]. The vast majority of salivary gland neoplasms arise in the major salivary glands, particularly the parotid gland. Consequently, oncocytomas of minor salivary glands account for a very small percentage of all salivary gland tumors. Their rarity makes accurate diagnosis and management challenging [ 7 ]. Oncocytomas of minor salivary glands typically present as slow-growing, painless masses [ 10 ]. An exhaustive literature review revealed 20 cases of oncocytoma occurring in minor salivary glands. Out of the 20 reported cases 12 were reported in females and 8 in males. The mean age of oncocytomas of minor salivary gland is 51.6 and intra orally the most common site is hard palate followed by buccal mucosa, oropharynx, and soft palate alveolus and retro molar area (Table 2). Histologically, these tumors are characterized by a well-circumscribed mass composed of large polyhedral cells (oncocytes) with abundant eosinophilic granular cytoplasm [ 5 - 6 , 9 - 11 ]. The nuclei of these cells are generally large and hyperchromatic [ 11 ]. These cells usually have granular cytoplasm due to the presence of abundant mitochondria [ 11 - 12 ]. Ultrastructural studies of parotid gland oncocytomas have shown that the cells contain large and morphologically altered mitochondria [ 5 , 13 - 15 ]. The diagnosis of oncocytoma requires the assessment of the tumor extent with panoramic radiography or magnetic resonance imaging (MRI) along with proper histopathological evaluation [ 7, 11, 16- 17 ]. The differential diagnoses of oncocytomas include Warthin’s tumor, acinic cell carcinoma, metastatic renal cell carcinoma, and mucoepidermoid carcinoma [ 11 ]. Warthin’s tumor usually shows bilayered epithelium supported by lymphatic tissue with well-developed germinal centers, which is missing in oncocytomas; acinic cell carcinomas show an infiltrative pattern; metastatic renal cell carcinomas show organoid pattern arrangement, which was absent in our case; and mucoepidermoid carcinomas show mucous cells, intermediate cells, and epidermoid cells with dysplastic features [ 18 - 20 , 22 ]. The granules in salivary gland oncocytoma can be identified on light microscopic examination with a phosphotungstic acid hematoxylin (PTAH) stain and by a combination with Periodic Acid Schiff stain [ 22 ]. There is a paucity of literature available regarding the use of immunohistochemical markers in the minor salivary glands oncocytomas of oral cavity; this limitation has prompted us to use markers in the present case. 

**Table 1:** Hallmark features of oncocytes [1, 7, 15]

Oncocytes
1. Special type of epithelial cells
2. Larger in size than normal cells
3. Microscopically the cells having mitochondria rich, dense cytoplasm containing acidophilic granules
4. The pathogenesis of oncocytes includes various factors i.e. metaplastic changes, inflammation, activation of oncogene, degenerative cellular changes, and adaptive mechanism
5. Oncocytic lesions include oncocytosis, oncocytoma and oncocytic carcinoma
6. Other lesions may show oncocytes include pleomorphic adenoma, Warthin’s tumor, adamantinoma, and adenoid cystic carcinoma

**Table 2:** A review of oncocytomas reported in minor salivary glands

S.NO	Author	Age	Gender	Site
1	Ahlbom [4]	59	Female	Hard palate
2	Miyoshi *et al*. [5]	53	Female	Hard Palate
3	Sato and Watanabe [6]	23	Female	Hard palate
4	Jalisi [10]	42	Female	Oropharynx
5	Crocker *et al*.[19]	77	Female	Hard Palate
6	Hung [15]	27	Male	Hard palate
7	Matsuda *et al*. [12]	53	Female	Buccal mucosa
8	Kohno [20]	59	Male	Hard palate
9	Hayashi *et al*.[18]	64	Male	Soft Palate
10	Hayashi *et al*.[18]	62	Male	Hard palate
11	Regezi *et al*. [14]	63	Female	Buccal mucosa
12	Chau and Radden [9]	58	Male	Buccal mucosa
13	Damm *et al*. [23]	73	Female	Buccal mucosa
14	Kochhar *et al*. [19]	45	Female	Hard palate
15	Kanazava *et al*. [8]	32	Female	Buccal mucosa
16	Camara *et al*. [16]	71	Male	Buccal mucosa
17	Yilmaz *et al*. [21]	72	Male	Maxillary posterior alveolus
18	Palakshappa *et al*. [18]	32	Female	Retro molar area
19	Majumdar BA *et al*. [7]	53	Female	Lingual aspect of left mandible
20	Shahi S *et al*.[14]	14	Male	Buccal mucosa
21	Present case	31	Male	Hard palate

Immunohistochemical features of minor salivary gland oncocytomas are underdocumented. While the morphological features of oncocytomas are generally distinct under the microscope, several other salivary gland tumors can exhibit similar characteristics, posing diagnostic challenges. Immunohistochemical examination utilizes antibodies that specifically bind to certain cellular proteins, acting as "molecular fingerprints" that can distinguish between different tumor types [ 21 - 22 ]. Distinguishing oncocytomas from other eosinophilic salivary gland tumors, such as acinic cell carcinoma, oncocytic carcinoma, and granular cell tumors, can be challenging. Immunohistochemical examination helps highlight specific markers unique to these different entities. Cytokeratins are intermediate filament proteins present in epithelial cells. Oncocytomas typically show broad-spectrum cytokeratin positivity, confirming their epithelial origin. CK7 is often strongly positive, aiding in differentiating them from granular cell tumors, which are typically negative for CK7. In contrast, CD117 is significantly expressed in certain aggressive salivary gland cancers like adenoid cystic carcinoma, making it a valuable marker for differentiating these tumors from oncocytoma [ 18 ]. The present case showed positive expression of neoplastic cells for CK-7 and negative expression for CD -117.

Few recent studies have shown that BSND protein is a good potential marker to differentiate oncocytoma and Warthin’s tumor from other oncocytoid tumors particularly oncocytic mucoepidermoid carcinoma [ 23 - 24 ].

Complete surgical excision is the treatment of choice for minor salivary gland oncocytomas; in parotid gland oncocytomas, superficial or complete parotidectomy may be required [ 11 , 20 , 23 ]. The present case was also treated with the complete surgical removal; no sign of recurrence was noted.

To report this case, informed consent of the patient was obtained.

## Conclusion

Minor salivary gland oncocytomas are rare, asymptomatic tumors with favorable prognosis and low recurrence rate; however, a proper diagnosis is required to rule out the possibility of malignant tumors like metastatic renal cell carcinoma, acinic cell carcinoma, and mucoepidermoid carcinoma in order to prevent the aggressive mode of treatment. Immunohistochemical staining can be used to confirm the diagnosis. Oncocytes typically stain positively for mitochondrial markers. Differential diagnosis includes other salivary gland tumors with oncocytic features, such as Warthin's tumor and mucoepidermoid carcinoma with oncocytic differentiation.
